# The status and trends of mitochondrial dynamics research: A global bibliometric and visualized analysis

**DOI:** 10.1007/s10863-023-09959-6

**Published:** 2023-02-21

**Authors:** Zijian Guo, Zehua Wang, Zhenzhong Gao, Tengda Feng, Yingjie Gao, Zhiwen Yin, Zui Tian, Yang Liu, Xingjia Mao, Chuan Xiang

**Affiliations:** 1 Department of Orthopedic, The Second Hospital of Shanxi Medical University, Taiyuan, China; 2 Department of Basic Medicine Sciences, and Department of Orthopaedics of Sir Run Run Shaw Hospital, Zhejiang University School of Medicine, Hangzhou, 310058 China

**Keywords:** Mitochondrial dynamics, Bibliometrics, Visualized analysis

## Abstract

**Background:**

Mitochondria are remarkably dynamic organelles encapsulated by bilayer membranes. The dynamic properties of mitochondria are critical for energy production.

**Aims:**

The aim of our study is to investigate the global status and trends of mitochondrial dynamics research and predict popular topics and directions in the field.

**Methods:**

Publications related to the studies of mitochondrial dynamics from 2002 to 2021 were retrieved from Web of Science database. A total of 4,576 publications were included. Bibliometric analysis was conducted by visualization of similarities viewer and GraphPadPrism 5 software.

**Results:**

There is an increasing trend of mitochondrial dynamics research during the last 20 years. The cumulative number of publications about mitochondrial dynamics research followed the logistic growth model $\mathrm{f}(x)=\mathrm{a}/\left[1+{e}^{(b-cx)}\right]$. The USA made the highest contributions to the global research. The journal *Biochimica et Biophysica Acta (BBA)—Molecular Cell Research* had the largest publication numbers. Case Western Reserve University is the most contributive institution. The main research orientation and funding agency were cell biology and HHS. All keywords related studies could be divided into three clusters: “Related disease research”, “Mechanism research” and “Cell metabolism research”.

**Conclusions:**

Attention should be drawn to the latest popular research and more efforts will be put into mechanistic research, which may inspire new clinical treatments for the associated diseases.

## Introduction

**Fig. 1:**
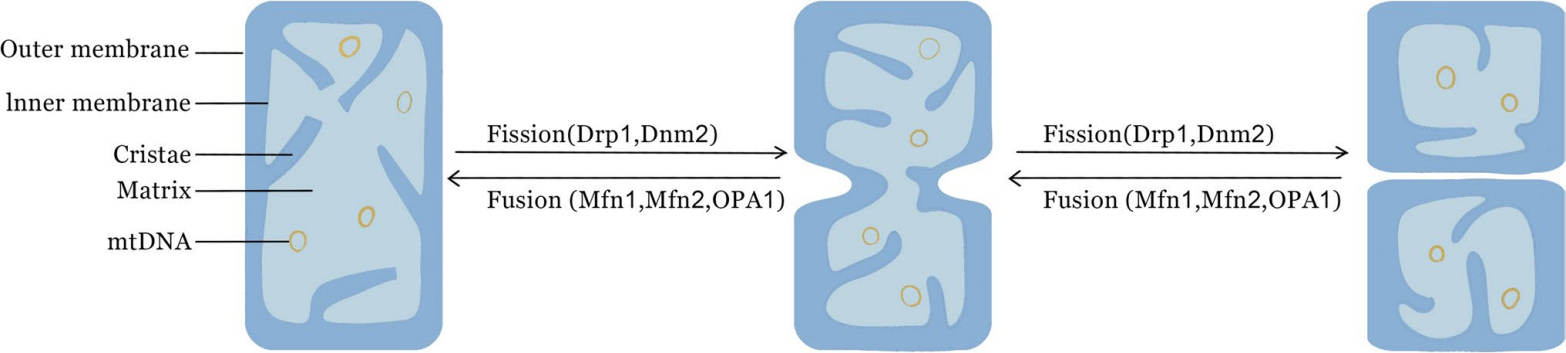
Schematic diagram of mitochondria and mitochondrial dynamics. Major components of mitochondria include outer membrane, inner membrane, cristae, matrix and mtDNA. Mitochondrial fusion is mediated by Mfn1, Mfn2 and OPA1. Mitochondrial fission is mediated by Drp1, Dnm2

Mitochondria, regarded as the “powerhouse” of eukaryotes, produce the energy needed for cell metabolism by oxidative phosphorylation (OXPHOS) (Kamer and Mootha 2015; Liu and Ho 2018). Mitochondria are remarkably dynamic organelles encapsulated by bilayer membranes, and the dynamic characteristics include mitochondrial fusion, mitochondrial fission and mitophagy (Chan 2020). Various physiological processes and metabolic regulations of cell are associated with mitochondrial dynamics (Wai and Langer 2016), such as autophagy, programmed celldeath, redox signaling, calcium homeostasis, innate immunity and stem cells reprogramming (Rambold and Pearce 2018; Tilokani et al. 2018). Mitochondrial fusion is the amalgamation of multiple mitochondria into one. The fusion of the external and interior membranes are mediated by mitofusion1 (Mfn1), mitofusion2 (Mfn2), and optic atrophy 1 (OPA1), respectively (Mao et al. 2020a). In contrast, the separation of mitochondria into two or more separate ones is known as mitochondrial fission. Dynamin-related protein 1 (Drp1) and the classical dynamin 2 (Dnm2) are the major mediators of mitochondrial fission (Lee et al. 2016; Pagliuso et al. 2018) (Fig. 1). For the normal function of cells, the balance between mitochondrial fusion and fission is essential, and this helps control the shape of the mitochondria (Duvezin-Caubet et al. 2006; Ishihara et al. 2006), exchange of content (Liu et al. 2009; Chen et al. 2010), maintenance of mitochondrial DNA (mtDNA) (Pagliuso et al. 2018; Cogliati et al. 2013) and clearance of damaged mitochondria (Kandul et al. 2016; Yamashita et al. 2016). Numerous human disorders, including Charcot-Marie-Tooth disease type 2A (CMT2A) (Züchner et al. 2004; Stuppia et al. 2015), Multiple symmetric lipomatosis (Rocha et al. 2017; Capel et al. 2018), Dominant optic atrophy (Chun and Rizzo 2017), Microcephaly and Optic atrophy (Waterham et al. 2007), have been linked to defects and damage in mitochondrial dynamics. Therefore, the mechanistic study of mitochondrial dynamics is gaining more and more attention in recent years, and treatments through mitochondrial pathway for related diseases are going to be a new clinical strategy (Mao and Wang 2020). However, studies on qualitative and quantitative characteristics of global research of mitochondrial dynamics are limited. Evaluation of the current status and trends of mitochondrial dynamics research and predicting promising popular topics and directions in the field are more essential.

A key measure of research contribution is publication, which is a component of scientific research (Wei et al. 2020). It has been acknowledged that using bibliometrics and graphical mapping together is a useful method for evaluating scientific advancement (López-Muñoz et al. 2015; Tijssen and Winnink 2016). Bibliometric analysis, which may be used to evaluate the changes in the research community over time both statistically and qualitatively, was used to examine data from online literature databases and metrology features (Ekinci et al. 2015). Through bibliometric analysis, comparisons between the contributions of academics, journals, organizations, and nations could be made (Pu et al. 2016). Additionally, clinical guidelines and policymaking both use bibliometric analyses (Avcu et al. 2015). Bibliometric analysis and visualization of mitochondrial dynamics-related studies will provide basic knowledge and hotspots for mitochondria-related research, and predict trends and cutting-edge research directions in the field of mitochondrial dynamics.Moreover, efficient analysis has been applied successfully to make studies more intuitional, including exosomes (Wang et al. 2019), celiac disease (Demir and Comba 2020), coronavirus research (Mao et al. 2020b), curcumin (Yeung et al. 2019), and infect diseases (Sweileh 2020). The aim of our study is to assess the current status and trends of global mitochondrial dynamics research, which will be presented in two parts. The first part is a multidimensional presentation of research results in the field over the past 20 years through a bibliometric approach, including an assessment of the quantity and quality of publications worldwide, as well as bibliographic coupling analysis, co-authorship analysis, co-citation analysis and co-occurrence analysis, in order to analyze current research hot topics and future research trends in the field. Finally, the bibliometric results are analyzed and summarized to lay the foundation for the global mitochondrial dynamics research and development.

## Materials and methods

### Date source

The Web of Science (WOS) Core Collection, which is regarded as the best resource for bibliometrics (Zhai et al. 2018; Wang et al. 2018), was searched for complete bibliometric information and the SCI-EXPANDED, SSCI, A&HCI, and ESCI citation index database (Aggarwal et al. 2016).

### Search strategy

The earliest relevant literature was published in 1966, but in order to collect all publications on mitochondrial dynamics in the last 20 years, the dataset from January 2002 to December 2021 was obtained from the WOS Core Collection. Theme words for searching were referred to MESH terms from PubMed (Shen et al. 2019), and the search term was as follows: ((TS = "Mitochondrial Dynamics") OR (TS = "Mitochondrial Dynamic") OR (TS = "Dynamic of Mitochondria") OR (TS = "Mitochondrial Fission and Fusion") OR (TS = "Mitochondrial Fusion and Fission")) AND (Language = English) AND (Document type = Articles OR Reviews). The information about publications including research orientation, institutions, and funding were ameliorated by the data in the WOS (Shen et al. 2018; Zhang et al. 2020a).

### Data collection

**Fig. 2:**
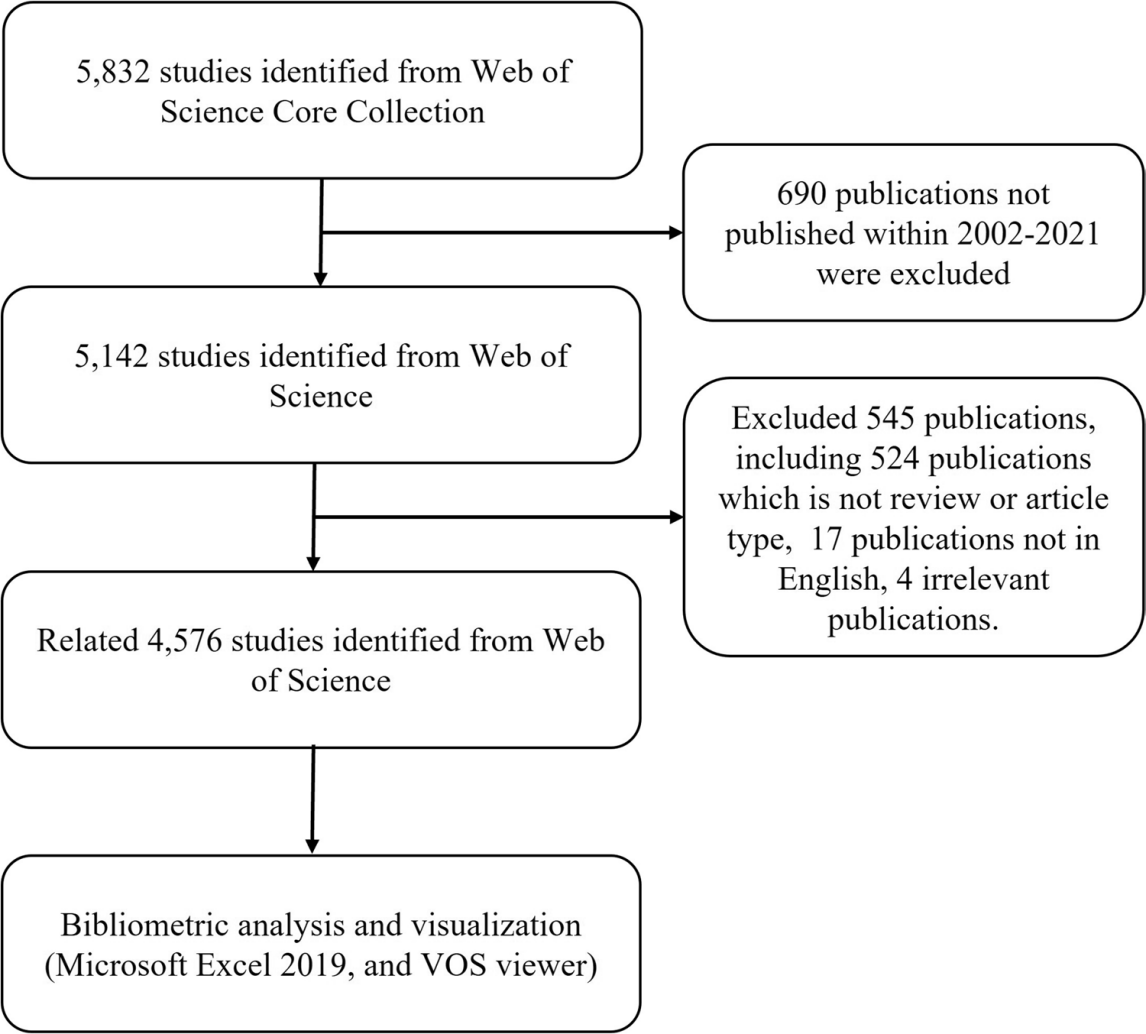
Flowchart for search and inclusion and exclusion of publications

The publications that were downloaded from WOS were saved as TXT files and imported into Microsoft Excel 2019 along with the titles, years of publication, names of authors, nationalities, H-index, affiliations, keywords, names of publishing journals, abstracts of each record, and citations within the publications (Chen et al. 2020a). Any issue had been resolved by consensus-building among specialists after consultation. Both document retrieval and download were completed within June 16, 2022 (Fig. 2).

### Bibliometric and visualized analysis

By utilizing bibliometric theory to examine relevant literature using mathematical and statistical methods, bibliometric analysis has become a crucial tool for comprehensive study and research across many scientific fields (Zou et al. 2018). Bibliometric analysis can not only quantitatively analyze the distribution of research areas in publications, as well as analyze the relationships and clustering characteristics among them, but also compare the relevant research contributions of different authors, countries, journals, and institutions, in addition to describing and predicting future research directions, and this analysis method has a crucial role in analyzing relevant research hotspots and predicting research trends (Ma et al. 2020; Zhang et al. 2020b; Guler et al. 2016). This method has been studied in the fields of digestive system diseases (Zhang et al. 2020c), cancer, and neurological diseases.Basic characteristics of eligible publications, which were mentioned previously, were described through the intrinsic function of WOS. The H-index was recommended as an improvement over existing bibliometric indices for determining the significance of scientific research (Bornmann and Daniel 2009). The index of H indicates that H of a scientist's or a nation's publications have been referenced in other publications at least H times each (Hirsch 2005), which takes into account both the total number of publications and the average number of citations per publication (Bertoli-Barsotti and Lando 2017). The Journal Citation Reports of 2021 provided the impact factors (IF) for all journals.

### Analytical methods

The logistic growth model $\mathrm{f}(x)=\mathrm{a}/\left[1+{e}^{(b-cx)}\right]$ was used to model the cumulative volume of documentation because of its great fitness and ability to predict the future trends (Bagley et al. 2001; Zhao et al. 2018), where x represents the year and f(x) is the cumulative volume of papers by year. All data are presented in Microsoft Excel 2019 (Redmond, WA, USA) and displayed in tables or charts using Excel functions. The data, including the time trend of the number of publications, the number of publications from various institutions, funding agencies, and research orientations, total citation frequency, average citation frequency, and H-index, were analyzed using GraphPadPrism 5 (GraphPad Software Inc., CA, USA) (Ma et al. 2021). A software program called VOS viewer (Leiden University, Leiden, Netherlands) was used to visually analyze publications and perform analyses on co-authorship, co-citation, and co-occurrence (Eck and Waltman 2010).

## Results

### Analysis of global publications

**Fig. 3:**
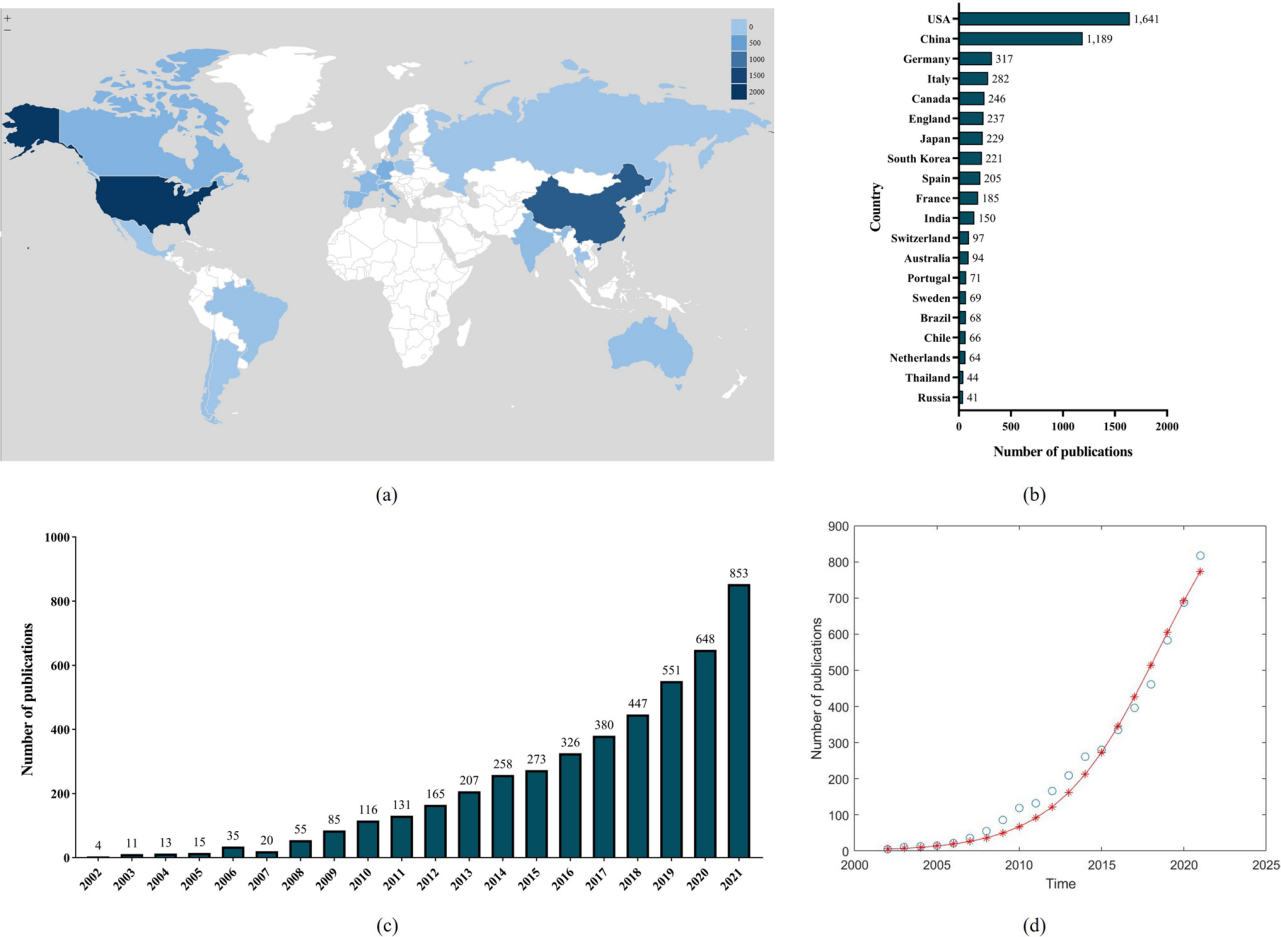
Global trends and contributed countries on mitochondrial dynamics research. (**a**) World map showing the distribution of mitochondrial dynamics research, in which the different color depths represent the different numbers of publications in different countries. (**b**) The sum of publications related to mitochondrial dynamics research from 25 countries or regions. (**c**) The annual number of publications related to mitochondrial dynamics research in the past 20 years. (**d**) Model fitting curves of growth trends of accumulated number of publications on mitochondrial dynamics research

**Fig. 4:**
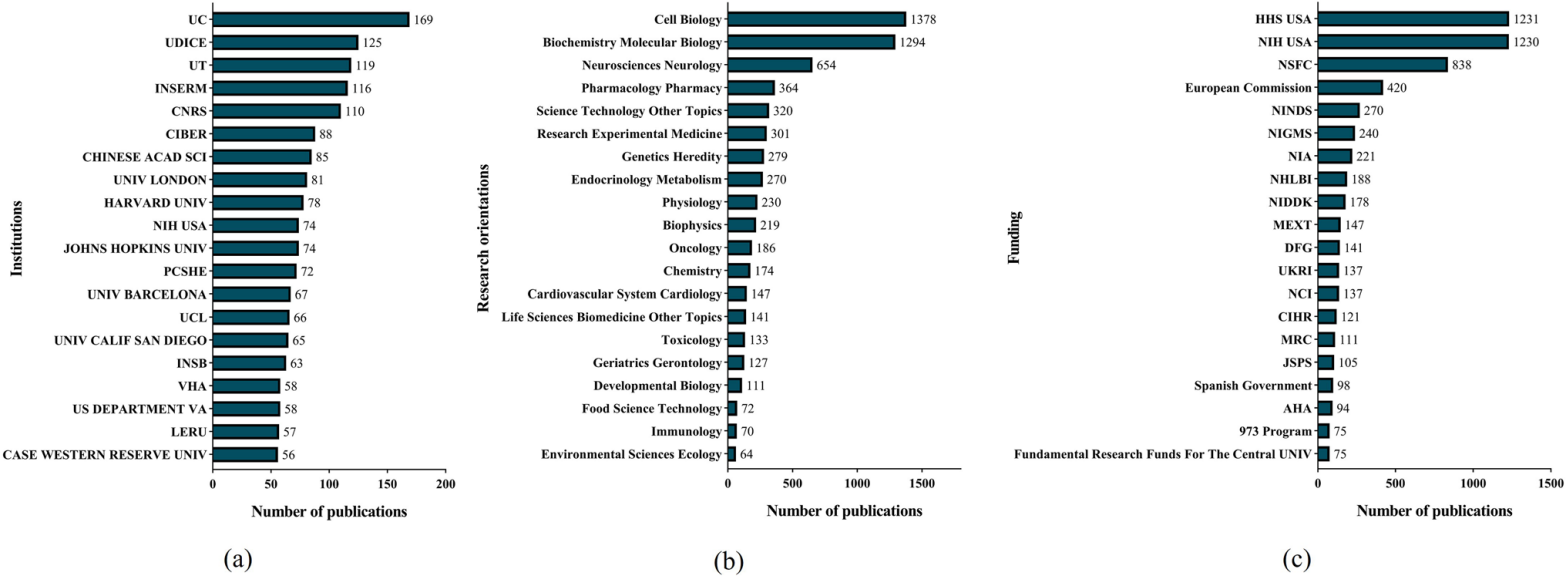
Institutions, research orientations and funding. (**a**) The most contributive institution with the most publications in mitochondrial dynamics research. (**b**) The most popular research orientation about mitochondrial dynamics. (**c**) The major contributive funds for mitochondrial dynamics

Variations in the volume of academic articles on a particular study subject are a key marker of the direction of progress. Understanding the research level and future trajectory is aided by multivariate statistical analysis and plotting the volume of publications over time. A total of 4576 publications from 2002 to 2021 were derived from WOS database according to the search criteria. Most research was published in the last 10 years (2012–2021, 4,195, 89.5%). An increasing trend of global publications during the last 20 years was found, which shows the relative research of mitochondrial dynamics will be more and more concerned (Fig. 3c). Furthermore, Fig. 3d showed the logistic regression model fitting curve $f\left(x\right)=\frac{1695.0406}{\left[1+164.4439{e}^{-0.2327x+465.4103}\right]}$ of the number of publications on mitochondrial dynamics research in the future per year. According to publications, the top 20 productive countries were listed in the Fig. 3b. USA (1,641; 35.86%) with the most number was the most contributor, followed by China (1,189; 25.98%), Germany (317; 6.93%), Italy (282; 6.16%) and Canada (246; 5.38%), Fig. 3a displays the top 25 nations in terms of research contributions to mitochondrial dynamics; the deeper the hue, the greater the number of publications. These economically successful or rapidly developing nations, as might be expected, place a high priority on scientific research. When it comes to the most contributive institutions, research orientations and funding, the ranking from WOS database were as followed respectively (Fig. 4): League of European Research Universities (344; 7.52%), University of California System (169; 3.69%) and Udice French Research Universities (125; 2.73%) and were listed in the top 3, and Cell Biology (1,378; 30.11%), Biochemistry Molecular Biology (1,294; 28.28%) and Neurosciences Neurology (654; 14.29%) were listed in the top 3, and Health and Human Servises (HHS) (1,231; 26.90%), National Institutes of Health (NIH) (1,230; 26.88%) and National Nature Science Foundation of China (NSFC) (838; 18.31%) were listed in the top 3.

### Quality of publications of different countries

**Fig. 5:**
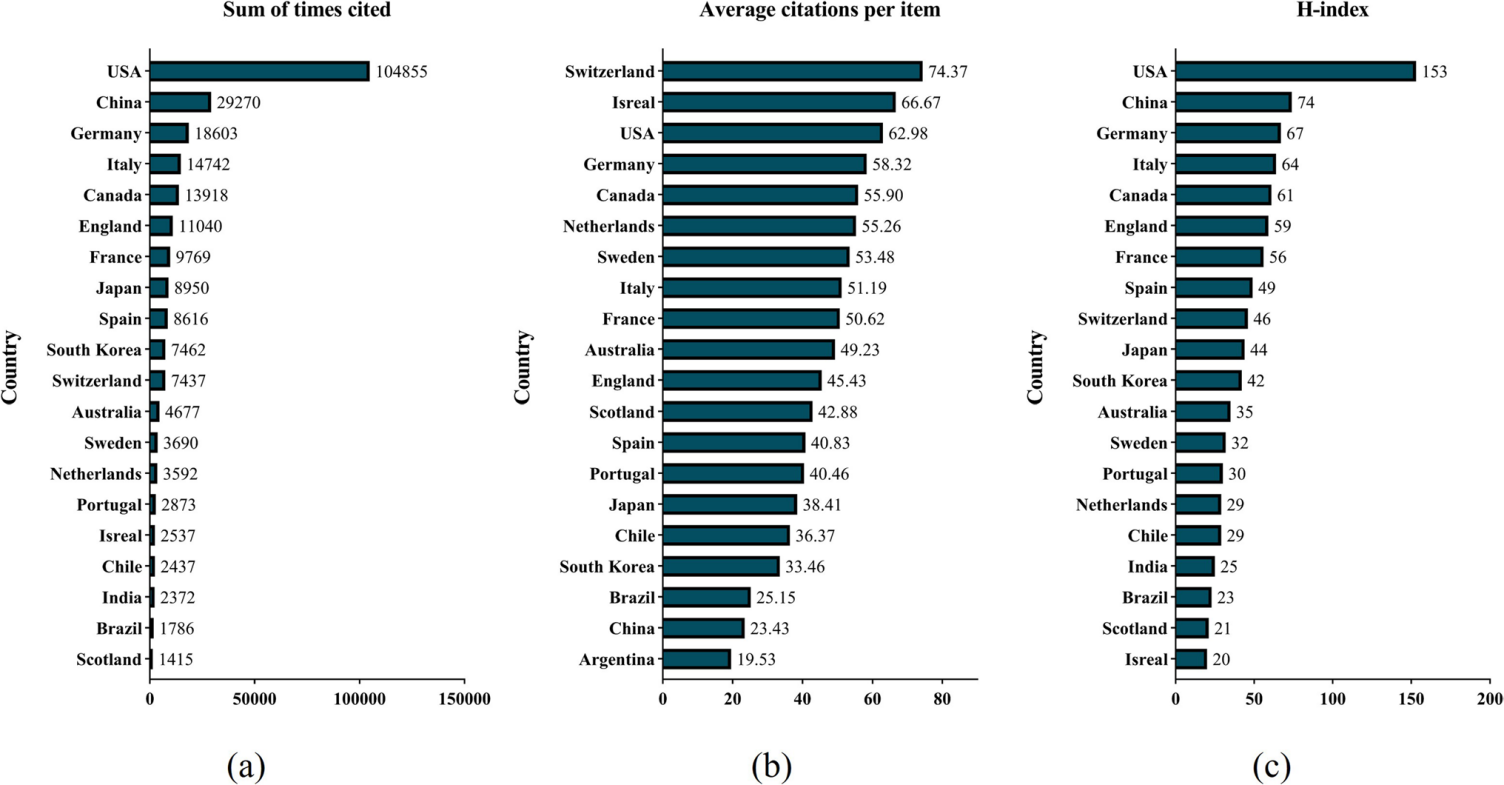
Citation frequency and H-index levels of different countries. (**a**) The total citations for mitochondrial dynamics research publications from different countries. (**b**) The average citations per paper for publications from the different countries. (**c**) The H-index of publications in the different countries

In regards to WOS database analysis, we tallied the total citations, average citations and H-index of each country (Fig. 5). United States papers received the most citations (104,855), followed by those from China (29,270), Germany, and Germany (18,603). Additionally, papers from the USA had the highest H-index at the time (153). China ranked second in H-index (71), followed by Germany (67), Italy (64) and Canada (61). While the top 5 countries in average citation frequency were Switzerland (74.37), Israel (66.67), USA (62.98), Germany (58.32) and Canada (55.90).

### Bibliographic coupling analysis

**Fig. 6:**
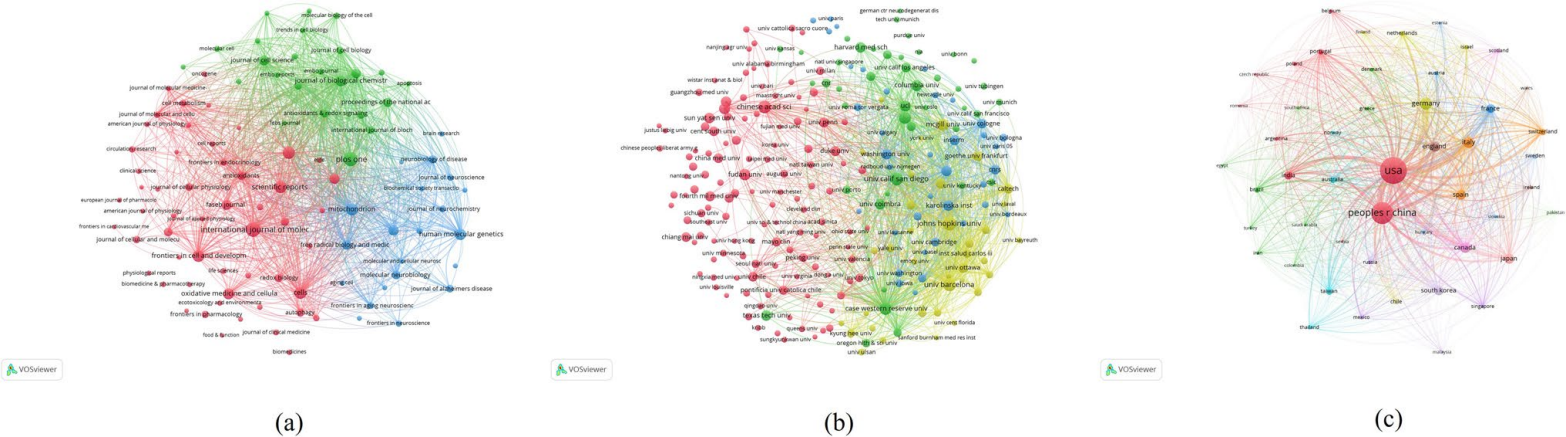
Bibliographic coupling analysis of global research about mitochondrial dynamics. (**a**) Mapping of the identified journals on mitochondrial dynamics research. (**b**) Mapping of the 241 institutions on mitochondrial dynamics research. (**c**) Mapping of the countries on mitochondrial dynamics research. The line between two points in the figure represents that two journals/institutions/countries had establish a similarity relationship. The thicker the line, the closer the link between the two journals/institutions/countries

In Fig. 6, each node represents a journal, an institution and a country, and the nodes’ size represents the power of total link strength (TLS). The links between nodes represent the associations where the greater thickness of the link (namely link strength) means the closer correlation, which is also utilized in co-authorship, co-citation, and co-occurrence analyses. Based on papers with the minimum number of documents of a journal more than 10, 99 journals were shown in Fig. 6a in accordance with total link strength (TLS) and the size of sphere representing the power of journal in the research field of mitochondrial dynamics. The top 5 journals with the greatest total link strength were as follows: *Biochimica et Biophysica Acta (BBA)—Molecular Cell Research* (Impact Factor, IF = 4.739; 2021; TLS = 152,012 times), *Mitochondrion* (IF = 4.160; 2021; TLS = 145,618 times),*International Journal Of Molecular Sciences* (IF = 5.923; 2021; TLS = 122,979 times), *Antioxidants & Redox Signaling* (IF = 8.401; 2021; TLS = 122,219 times) and *Cells* (IF = 6.600; 2021; TLS = 111,898 times) (Table 1). Also shown in Fig. 6a is the analysis of bibliographic sources clusters. Cluster 1 (red) is cell metabolism and contains 54 journals with a total of 1266 publications. Among them *International Journal of Molecular Sciences* (USA; 96 documents), *Cells* (Switzerland; 62 documents), *Frontiers In Cell and Developmental Biology* (Switzerland; 61 documents), *Biochemical and Biophysical Research Communications* (USA; 66 documents) and *Antioxidants* (Switzerland; 36 documents) are the five most talked-about journals. These include studies on mitochondrial dynamics in cellular metabolism, redox and other biochemical and physiological aspects. Cluster 2 (green) is mechanistic research and contains 24 journals with 609 publications, of which *Biochimica et Biophysica Acta-Molecular Cell Research* (Netherlands; 42 documents), *Antioxidants & Redox Signaling* (USA; 31 documents), *Plos One* (USA; 91 documents), *Cellular and Molecular Life Sciences* (Switzerland; 24 documents) and *Journal of Biological Chemistry* (USA; 53 documents) are the five most talked-about journals. These contain studies on specific mechanisms of mitochondrial dynamics regulation and the links and mechanisms of influence with cell biology. Cluster 3 (blue) is disease research and contains 21 journals with a total of 523 publications. Among them M*itochondrion* (England; 62 documents), *Neurobiology of Disease* (England; 31 documents), *Biochimica et Biophysica Acta-Molecular Basis of Disease* (Netherlands; 49 documents), *Human Molecular Genetics* (England; 58 documents) and *Free Radical Biology and Medicine* (USA; 41 documents) are the five most talked-about journals. The role of mitochondrial dynamics in the development of disease and the study of mitochondrial dynamics as a therapeutic disease target are analyzed in these studies. Based on papers with the minimum number of documents of an organization more than 10, 241 institutions are depicted in Fig. 6b. Case Western Reserve University (TLS = 336,543 times) ranked the first, followed by Universitat de Barcelona (TLS = 243,701 times), The University California, San Diego (TLS = 287,853 times), The Johns Hopkins University (TLS = 266,374 times) and The University of Padua (TLS = 258,527 times). The top 5 countries based on papers with the minimum number of documents of a country more than 5 were shown in Fig. 6c, including USA (TLS = 4,748,998times), China (TLS = 2,286,839 times), Germany (TLS = 1,201,298 times), Italy (TLS = 1,115,597 times) and Canada (TLS = 975,448 times).

**Table 1:** The 10 most talked-about journals

Journals	Documents	Total link strength	Country	IF(2021)	Citations
*Biochimica et Biophysica Acta-Molecular Cell Research*	42	152,012	Netherlands	5.011	3358
*Mitochondrion*	62	145,618	England	4.534	1755
*International Journal of Molecular Sciences*	96	122,979	USA	6.208	1550
*Antioxidants & Redox Signaling*	31	122,219	USA	7.468	1814
*Cells*	62	111,898	Switzerland	7.666	1207
*Plos One*	91	110,767	USA	3.725	4275
*Frontiers in Cell and Developmental Biology*	61	102,125	Switzerland	6.081	921
*Cellular and Molecular Life Sciences*	24	98,331	Switzerland	9.207	966
*Journal of Biological Chemistry*	53	94,098	USA	5.486	3667
*Neurobiology of Disease*	31	91,398	England	7.046	1470

### Co-authorship analysis

**Fig. 7:**
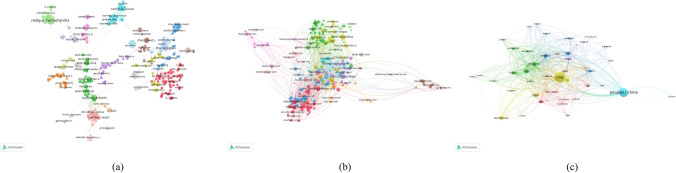
Co-authorship analysis of global research about mitochondrial dynamics. (**a**) Mapping of the 77 authors co-authorship analysis on mitochondrial dynamics research. (**b**) Mapping of the 241 institutions co-authorship analysis on mitochondrial dynamics research. (**c**) Mapping of the 49 countries co-authorship analysis on mitochondrial dynamics research. The size of the points represents the co-authorship frequency. The line between two points in the figure represents that two authors/institutions/countries had establish collaboration. The thicker the line, the closer the collaboration between the two authors/institutions/countries

In order to show the network of collaboration among the primary research authors, institutions, and nations in the study of mitochondrial dynamics, co-authorship analysis seeks to link things based on the number of co-authored papers they have produced. The KDM of co-authorship network of successful authors, institutions, and nations may be constructed and studied to help researchers identify partners for cooperation, research organizations build cooperative groupings, and nations fulfill the goal of academic exchanges. Authors, institutions and countries were analyzed via VOS viewer and 77 authors, 241 institutions and 49 countries were shown in Fig. 7. Chattipakorn, N (TLS = 85 times), Chattipakorn, SC (TLS = 84 times), Jaiwongkam, T (TLS = 69 times), Zhu, XW (TLS = 67 times) and Reddy, PH (TLS = 66 times), and were considered as the top 5 authors with the largest TLS. By the same way, the top 5 institutions with the TLS were as follows: Chinese Academy of Sciences (TLS = 86 times), The Johns Hopkins University (TLS = 72 times), The Baylor college of Medicine (TLS = 69 times), Case Western Reserve University (TLS = 63 times) and Centre National de la Recherche Scientifique (TLS = 63 times). Both authors and institutions were based on papers with the minimum number of documents of an author or organization more than 10. The top 5 countries with largest TLS based on papers with the minimum number of documents of a country more than 5 were USA (TLS = 898 times), China (TLS = 321 times), England (TLS = 305 times), Germany (TLS = 301 times), and Italy (TLS = 220 times).

### Co-citation analysis

**Fig. 8:**
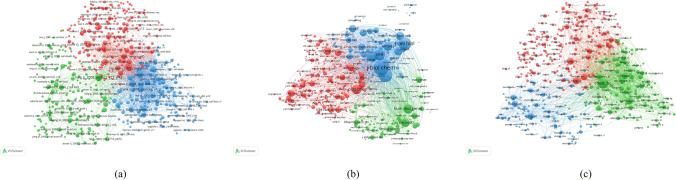
Co-citation analysis of global research about mitochondrial dynamics. (**a**) Mapping of co-cited references related to mitochondrial dynamics research. (**b**) Mapping of co-cited journals related to mitochondrial dynamics research. The points with different colors represent the cited references/journals. The size of the points represents the citation frequency. A line between two points means that both were cited in one paper/journal. A shorter line indicates a closer link between two papers/journals. (**c**) Mapping of the authors co-citation analysis on mitochondrial dynamics research. Points in the same color belong to the same research direction

Based on how often two items are referenced together, co-citation analysis seeks to ascertain how closely connected they are. From the large number of cited references, the key knowledge bases of the study topic may be quickly and easily determined using co-citation analysis. Furthermore, the relevance of publications can also be analyzed and excavated. Table 2 shows the top 10 most cited publications. Papers with the minimum number of citations of a cited reference more than 50 were analyzed using VOS viewer and 545 references were shown in Fig. 8a. The top 5 were as follows: *The Journal of Cell Biology*. 2003. 160(2): 189–200 (Chen et al. 2003). ( TLS = 21,293 times), *The EMBO Journal*. 2008. 27(2): 433–46 (Twig et al. 2008). (TLS = 19,809 times), *Molecular biology of the cell*. 2001. 12(8): 2245–56 (Smirnova et al. 2001). (TLS = 16,286 times), *Developmental cell*. 2001. 1(4): 515–25 (Frank et al. 2001). (TLS = 14,663 times) and *The Journal of biological chemistry*. 2007. 282:11,521–9 (Taguchi et al. 2007). (TLS = 14,448 times). Journals with at least 80 citations were analyzed through VOS viewer and there were 533 journals shown in Fig. 8b. Following are the top 5 journals with the most overall link strength: *The Journal of Biological Chemistry* (TLS = 1,636,817 times), *Proceedings of The National Academy of Sciences of The United States of America* (TLS = 1,380,705 times), *Journal of Cell Biology* (TLS = 1,169,260 times), *Nature* (TLS = 959,053 times) and *Cell* (TLS = 912,166 times). Also Fig. 8b shows the similarity between journals based on two or more journals cited by the same publication constituting a classification of journals into three clusters (Moya-Anegon et al. 2006). The first cluster (red colour) is devoted to the study of mitochondrial dynamics in biochemistry as well as molecular biology. In total, it contains 285 journals with 118,725 citations. Within this cluster, *Plos One* (USA; 617, 408 citations), *Cell Metabolism* (USA; 438, 159 citations), *Autophagy* (USA; 341, 247 citations), *Cell Death and Differentiation* (England; 319, 181 citations), *Biochemical and Biophysical Research Communications* (USA; 310, 756 citations) are the top 5 most popular journals. In this cluster are mainly included studies on the specific molecular mechanisms of mitochondrial dynamics in cellular metabolism, cellular physiological functions, etc. The second cluster (green colour) is devoted to the study of mitochondrial dynamics in neurology. In total, it contains 125 journals with 61,665 citations. Among them *Human Molecular Genetics* (England; 722, 622 citations), *Journal of Neuroscience* (USA; 605, 367 citations), *Nature Genetics* (USA; 324, 957 citations), *Journal of Neurochemistry* ( England; 316, 367 citations), and *Neuron* (USA; 302, 660 citations) are the five most popular journals. This cluster contains mainly research on the pathogenic role of mitochondrial dynamics in the neurology of stroke, Parkinson's disease, Alzheimer's disease, and neuromuscular diseases, as well as therapeutic targets. The third cluster (blue colour) is devoted to mitochondrial dynamics research in cell biology and immunology. A total of 120 journals with 136,900 citations are included. Among them, *Journal of Biological Chemistry* (USA; 1,636,817 citations), *Proceedings of the National Academy of Sciences of the United States of America* (USA; 1,380,705), *Journal of Cell Biology* (USA; 1,169,260 citations), *Nature* (England; 959,053 citations), *Cell* (USA; 912, 166 citations) are the five most popular journals. This cluster contains mainly studies on mitochondrial dynamics in cell proliferation, apoptosis, metabolism, and genetics.

**Table 2:** Top 10 most cited documents

Rank	Auther	Title	Citations	Journal	Year
1	Youle RJ et al	Mitochondrial fission, fusion, and stress	1821	*Science*	2012
2	Jeong SY et al	The role of mitochondria in apoptosis	1386	*BMB Reports*	2008
3	McBride HM et al	Mitochondria: more than just a powerhouse	1233	*Current Biology*	2006
4	Westermann B	Mitochondrial fusion and fission in cell life and death	1226	*Nature Reviews Molecular Cell Biology*	2010
5	Martinou JC et al	Mitochondria in apoptosis: bcl-2 family members and mitochondrial dynamics	1039	*Developmental Cell*	2011
6	Chen H et al	Mitochondrial dynamics-fusion, fission, movement, and mitophagy-in neurodegenerative diseases	990	*Human Molecular Genetics*	2009
7	Detmer SA et al	Functions and dysfunctions of mitochondrial dynamics	941	*Nature Reviews Molecular Cell Biology*	2007
8	Suen DF et al	Mitochondrial dynamics and apoptosis	899	*Genes & Development*	2008
9	Ashrafi G et al	The pathways of mitophagy for quality control and clearance of mitochondria	851	*Cell Death and Differentiation*	2013
10	Cipolat S et al	OPA1 requires mitofusin 1 to promote mitochondrial fusion	840	*Proceedings of the National Academy of Sciences of The United States of America*	2004

Papers with the minimum number of citations of an author more than 80 were identified and analyzed via VOS viewer and 434 authors were shown in Fig. 8c. The top 5 authors with the greatest total link strength were as follows: Chen HC (TLS = 84,477 times), Ishihara N (TLS = 42,421 times), Karbowski M (TLS = 40,103 times), Twig G (TLS = 39,612 times) and Youle, RJ (TLS = 29,806 times). It can also be seen in Fig. 8c that two authors share the same area of research if the cited author's publication is co-cited by one or more documents (Diez-Martin et al. 2021; Kim et al. 2016). Cluster 1 (red colour) studies are mainly about the role of mitochondrial dynamics in mitochondrial homeostasis and the connection with mitochondrial synthesis, mitophagy. The representative authors are Twigg, G (1,060 citations), Youle, RJ (1,025 citations), Chan, DC (934 citations). Cluster 2 (green colour) studies focus on the role played by mitochondrial dynamics in cell biology, such as proliferation and apoptosis. The representative authors are Chen, HC (84,477 citations), Ishihara, N (42,421 citations), and Karbowski, M (40,103 citations). Cluster 3 (blue colour) studies focus on the neurological aspects of mitochondrial dynamics, such as pathogenesis and therapeutic measures. The representative authors are Wang, XL (28, 859 citations), Reddy, PH (22, 659 citations), Manczak, M (16, 669 citations).

### Co-occurrence analysis

**Fig. 9:**
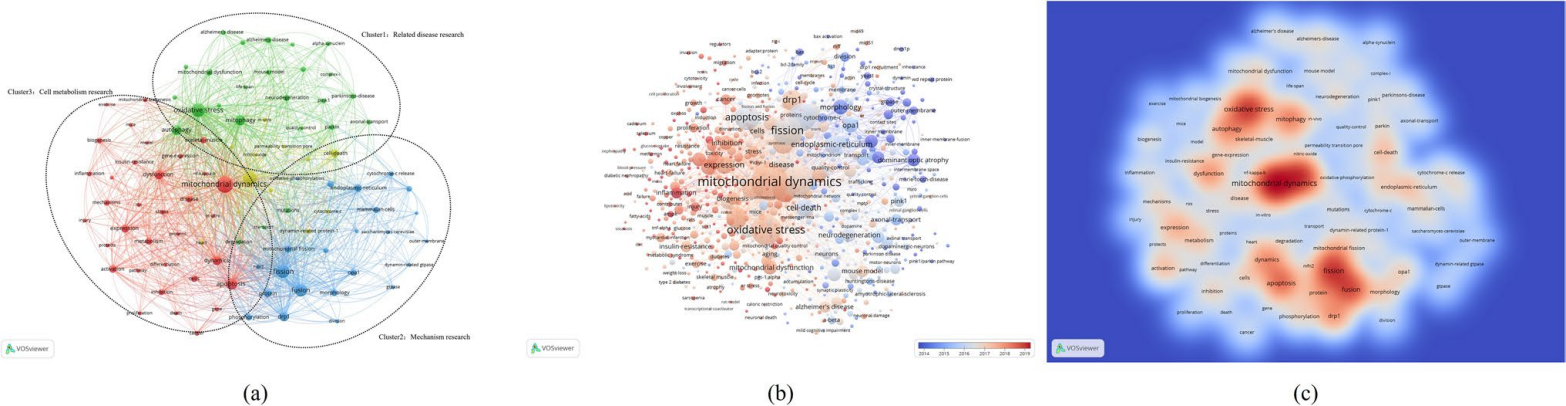
Co-occurrence analysis of global research about mitochondrial dynamics. (**a**) Mapping of keywords in the research on mitochondrial dynamics. The size of the points represents the frequency, and the keywords are divided into 3 clusters: “Related disease research” (upper in green), “Mechanism research” (right in blue) and “Cell metabolism research” (left in red). (**b**) Distribution of keywords according to the mean frequency of appearance. Keywords in blue appeared earlier than those in yellow and red colored keywords appeared later. (**c**) Density visualization map showed that “Fission”, “Fusion”, “Mitochondria”, “Oxidative stress”, “Mitophagy” and “Apoptosis” were the most relevant keywords related to mitochondrial dynamics in general

As shown in Fig. 9a, 94 identified keywords were classified into the 3 clusters: green cluster represents “Related disease research”, blue represents “Mechanism research” and red represents “Cell metabolism research”. The findings showed that the aforementioned 3 routes were among the most important sectors of mitochondrial dynamics. For the “Related disease research” cluster, the primary keywords were Aging, Alpha-Synuclein, Alzheimer-disease, Parkinsons-disease, amyloid-beta, mitochondrial dysfunction and oxidative stress. For the cluster of “Mechanism research”, frequently used keywords were Apoptosis, Cytochrome-c, Drp1, OPA1, Dynamin-related protein, Mitofusin 2, Mitochondrial fusion or fission. In the “Cell metabolism research” cluster, the main keywords were Activation, Autophagy, Metabolism, Mutation, Gene-expression and Degradation.

Table 3 shows the 15 keywords with the highest occurrence in mitochondrial dynamics studies. VOS viewer colored 778 keywords according to the average number of times they appeared in the total of 4689 articles (Fig. 9b). Blue indicates that the keywords occurred earlier, while red indicates that the keywords appeared later. Before 2012, most studies focused on “Saccharomyces-cerevisiae”, “Cytochrome-c release” and “Morphology”. The latest trends showed that “Mitofusin 2”, “Dynamin-related protein 1”, “Mitophagy”, “Biogenesis”,“Metformin”, “Inflammation”and “Mechanism” would be concerned more widely in the future.

**Table 3:** The main 15 key words of mitochondria dynamics

Rank	Key Words	Occurrences	Total Link Strength	Links
1	Mitochondrial dynamics	1644	7414	93
2	Mitochondria	1164	5671	93
3	Oxidative stress	1104	5436	93
4	Fission	1057	5982	93
5	Fusion	927	5280	93
6	Apoptosis	758	4062	93
7	Mitophagy	636	3753	93
8	Autophagy	549	3095	93
9	Dysfunction	544	2969	91
10	DRP1	534	3074	93
11	Dynamics	534	2801	92
12	Expression	389	1849	89
13	Protein	352	1749	89
14	Endoplasmic-reticulum	345	1884	92
15	Activation	344	1767	90

Papers with the minimum number of occurrences of a keyword more than 80 were identified and analyzed via VOS viewer. Density visualization map (Fig. 9c) was exported by VOS viewer. The times of occurrence of a key word was defined as the color of the area. The larger the tines, the warmer the color is. Red and blue are corresponding to the highest and lowest item densities, respectively. “Fission”, “Fusion”, “Mitochondria dynamics”, “Oxidative stress” and “Apoptosis” were the most relevant keywords related to mitochondrial dynamics in general.

## Discussion

### 1. Trends in publications of mitochondrial dynamics research

The present study purposes to assess mitochondrial dynamics research with visualized and bibliometric analysis, which can be applied to present the current status and make predictions in a certain research field. Dramatic progress in mitochondrial dynamics research especially in the last decade was analyzed in our study. Mitochondrial dynamics were first studied in single-celled organisms (Sedar and Porter 1955; Hermann and Shaw 1998) and in Drosophila (Watanabe and Williams 1953), and later expanded to human-related diseases and cells, as retrieved from the WOS database. Mitochondria had previously served as organelles for cellular energy production and fatty acid oxidation until researchers discovered that mitochondria can retain their own DNA (mtDNA) as well as transcription and translation (Friedman and Nunnari 2014), and have kinetic characteristics of fusion and breakage as well as mitophagy (Ruan et al. 2020). Since then researchers have collectively referred to the above three points as mitochondrial quality control (MQC) and have studied the complex MQC mechanisms to maintain mitochondrial functional and morphological integrity and the role they play in various diseases or targets for therapy (Xin et al. 2021), and mitochondrial dynamics-related studies have increased rapidly, as is consistent with our statistics and expectations. According to our analysis, 68 nations and regions published papers and reviews in the topic, and based on the available information, the number of publications in the upcoming years might be estimated. The results of our study also provide potential popularities in the field, which need further high-quality researches. Moreover, there will be more and more in-depth studies of mitochondrial dynamics and related articles will be published in the coming years.

### 2. Status and quality of global publications

Based on the results of the analysis about contributions of countries, USA was the most contributor to publications.Also, according to the search, the earliest study of mitochondrial dynamics in eukaryotes was published by the American researcher Pinkert,CA (Hermann and Shaw 1998), and was funded by NIH and HHS. This shows that the U.S. research in mitochondrial dynamics started earlier than other countries, and has a lot of experience and attention. This is consistent with the funding agency statistics, where 7 of the top 10 funding agencies and 71.69% of the top 10 funded publications are from the United States. In terms of total number of publications, total citation frequency and H-index, the United States contributes the most to global research. Although institutionally, only 2 U.S. institutions are among the top 10 institutions with the highest number of publications and only 21.90% of publications, the United States can still be considered a pioneer and leading country in the field of mitochondrial dynamics research. Switzerland has the highest average number of citations and is ranked 9th in the H-index, indicating that Switzerland is also an outstanding contributor to mitochondrial dynamics research. The top 5 related publications research areas are cell biology, biochemistry molecular biology, neurosciences, multidisciplinary sciences, and genetics heredity. However, Switzerland had the highest average number of citations and ranked 9th in H-index, which demonstrated that Switzerland was also an excellent contributor in mitochondrial dynamics research. In terms of publication number, the League of European Research Universitie, Cell Biology and United States Department Of Health Human Services ranked first in institutions, research orientations and funding agency, respectively. As a result of the number of related publications, there was a mismatch between H-index and average number of citations, the H-index looks more at the level of overall research in a country over time and can eliminate the impact of anomalous publications that may mislead researchers. Both were used for representing the quality of publications and the academic impact of a country. China came second in terms of overall publications, but its average citation frequency was 21st. There may have been a disparity between the amount and quality of publications since the Chinese academic grading system frequently emphasizes quantity over quality (Zhai et al. 2016). This policy prones researchers and doctors to publish articles more quickly, thereby the quality of studies was ignored. The quality of studies will significantly improve with the steady growth in financing for research in China to keep up with international publications in the subject.

When two works both reference a third work in their bibliographies, this is referred to as "bibliographic coupling.". By using bibliographical coupling analysis, we were able to establish a relationship of similarity between various publications in our study in terms of the journal, organization, and nation. *Biochimica et Biophysica Acta (BBA)—Molecular Cell Research*, *Mitochondrion, International Journal Of Molecular Sciences* and *Antioxidants & Redox Signaling* shown in Fig. 6a, may be the core journals of mitochondrial dynamics research. In the journal bibliometric coupling analysis, the journals were divided into three sections: cellular metabolism, basic research, and disease research, mainly based on the content of the research. Among them, journals in the area of mechanistic research had the highest number of publications and total link intensity.The aforementioned publications are more likely to report on the most recent scientific advancements in this field. Case Western Reserve University with the most TLS was regarded as the leader institution in mitochondrial dynamics research. There were 4 institutions from USA in the top 5 list, which was consistent with the strength of USA in the field. This implied that elevating a nation's academic standard required the establishment of world-class research institutes in a large and fundamental way. The authors depicted in Fig. 6c may have made the greatest contributions to the field, thus it is important to closely follow their future research and most recent publications to learn about the most recent developments in the field of mitochondrial dynamics. Co-authorship analysis was used to assess the cooperation between various authors, institutions, and nations, and the top findings with the highest overall link strength indicated that the writers, institutions, and nations were eager to cooperate. For example, Chattipakorn, N, Chinese Academy of Sciences and USA were the optimum choices for us to cooperate with. By quantifying the number of times a work was mentioned, co-citations analysis is used to gauge its significance. The present results indicated that the mechanism studies about mitochondrial dynamics had the greatest total frequency of citation and many meaningful references were provided. *The Journal of Cell Biology* was the journal with the highest citation frequency in the field and the achievement of Chen HC, Ishihara N and Karbowski M were widely recognized.

### 3. Focus of mitochondrial dynamics research

According to the co-occurrence analysis, popular topics and latest research directions were identified. The map of a co-occurrence network was shown in Fig. 9a by analyzing the keywords appearing in title and abstract from all included studies. Three research directions were observed from the co-occurrence network map, including “Related disease research”, “Mechanism research” and “Cell metabolism research”. Our study could make a further clear for the trends of future research, even though the results were consistent with the common sense in the field. In the center of the co-occurrence map, in addition to “mitochondrial dynamics”, other keywords such as “Fission”, “Fusion”, “Mitochondria”, “Oxidative stress” and “Apoptosis” were shown with higher total link strength (Fig. 9c). Under the influence of certain adverse factors, such as inflammation and mechanical damage, cells generate large amounts of oxygen radicals (ROS), which induce oxidative stress in mitochondria, with specific changes including mtDNA damage, loss of membrane potential, reduced energy production, increased membrane permeability, and release of pro-apoptotic factors such as cytochrome C (Cyt-C) and procaspases, which activate downstream caspases, thus inducing apoptosis and promoting cell injury (Lepetsos and Papavassiliou 2016). And mitochondrial dynamics can increase the resistance of mitochondria to injury. Mitochondrial kinetic content mainly includes mitochondrial fusion and breakage (Andreux et al. 2013). Mitochondrial fusion is divided into outer mitochondrial membrane fusion, which is mediated by Mfn1 and Mfn2, and inner mitochondrial membrane fusion, which is mediated by OPA1 (Cao et al. 2017). Mitochondrial fusion is essential during growth and development (Chen et al. 2003). Malfunctioning mitochondria within a cell can fuse with each other to maintain overall mitochondrial function and maintain a stable cellular energy supply (Olichon et al. 2003). In contrast, mitochondrial division is mainly mediated by Drp1 and classical Dnm2. Increased mitochondrial breakage is closely related to apoptosis, and it has been shown that inhibition of Drp1 expression can effectively inhibit mitochondrial division and slow down the process of apoptosis (Breckenridge et al. 2003). However, mitochondrial breakage also plays an important role in cell development and neural growth (Verstreken et al. 2005).

The overlay visualization map (Fig. 9b) was the same as the co-occurrence map but in color. Different colors represent different publishing years. Based on the results, “Mitofusin 2”, “Dynamin-related protein 1”, “Mitophagy”, “Biogenesis”,“Metformin”, “Inflammation”and “Mechanism” may become the next popular subjects in mitochondrial dynamics research. This is in line with the current hot trend of mitochondrial dynamics research. The relationship between mitochondrial dynamics and mitophagy has been gradually revealed in recent years that mitochondrial division activates mitophagy mediated by Serin/threonine-protein phosphatase and tensin homolog-induced kinase 1 (PINK1)/Parkin, and that Drp1 plays an important role in this reaction (Westermann 2010). This explains why mitophagy has become an emerging research hotspot for the study of mitochondrial dynamics. Meanwhile, the research in the mechanism of related diseases have been emerging widely in recent years. Abnormalities in the function of molecules related to mitochondrial dynamics have been shown to play an important role in certain diseases, for example, OPA1 and MFN2 mutations are causative factors in two neurodegenerative diseases (Amati-Bonneau et al. 2008), and muscle defects associated with neuromyelination have also been found in mice carrying OPA1 mutations (Alavi et al. 2009). Abnormal mitochondrial morphology was likewise found in senescent cells with altered expression profiles of molecules such as MFN1, MFN2, and OPA1 (Crane et al. 2010; Marzetti et al. 2017). Futhermore, the relevance of mitochondrial dynamics to ischemic diseases (Yu et al. 2020; Maneechote et al. 2019; Guan et al. 2019), renal diseases (Zhong et al. 2021; Chen et al. 2020b), immune-related diseases (Mills et al. 2017; Banoth and Cassel 2018) and cancer (Porporato et al. 2018; Zamorano-León et al. 2019), among others, has been studied.In addition, the mechanism of the treatment through mitochondrial pathway for related diseases may become a popular topic in the future.

### 4. Strengths and limitations

The Science Citation Index-Expanded Web of Science database was used to retrieve publications on mitochondrial dynamics research for our study. Bibliometric and visualized analysis, which was deemed bibliometric and visualized analysis, which was supposed to be reasonably objective and thorough, was used to evaluate the status and trends of mitochondrial dynamics research. However, some limitations in our study have to be mentioned here. Publications in non-English were excluded, resulting in language bias. Additionally, studies published in 2022 were not included in this study and recently published high-quality articles might not be highlighted as a result of low citation frequency until now. Moreover, the document type was limited to articles and reviews. Therefore, further research should address the latest studies and other non-English publications, and include other types of documents as complete as possible.

## Conclusion

The current study presented the global status and trends in mitochondrial dynamics research. The USA with the most contributions to the research plays a leading role in global research on mitochondrial dynamics. The quantity, caliber, and academic influence of publications from China varied significantly. The majority of papers of the study are found in *Biochimica et Biophysica Acta (BBA)—Molecular Cell Research*. Notably, in the upcoming years, a growing number of research regarding mitochondrial dynamics will be published. The study of mechanism research and cell metabolism research will be concerned and scholars focusing on “Mitofusin 2”, “Dynamin-related protein 1” and “Mitophagy” are likely to be pioneers in this field and conduct the direction of future studies, which may contribute to a new clinical treatment for the related disease associated with the imbalance of mitochondrial dynamics.

## Data Availability

The data from the review are available from the corresponding author on reasonable request.

## Abbreviations

*SCI-E*: Science Citation Index-Expanded
*WOS*: Web of Science
*OXPHOS*: Oxidative phosphorylation
*IF*: Impact factor
*TLS*: Total link strength
*Mfn1*: Mitofusion1
*Mfn2*: Mitofusion2
*OPA1*: Optic Atrophy 1
*Drp1*: Dynamin-related protein 1
*Dnm2*: Dynamin 2
*mtDNA*: Mitochondrial DNA
*CMT2A*: Charcot-Marie-Tooth disease type 2A
*KDM*: Knowledge domain map
*MQC*: Mitochondrial quality control
*ROS*: Oxygen radicals
*Cyt-C*: Cytochrome C
*PINK1*: Serin/threonine-protein phosphatase and tensin homolog-induced kinase 1
